# Exploring Burnt Area Delineation with Cross-Resolution Mapping: A Case Study of Very High and Medium-Resolution Data

**DOI:** 10.3390/s25103009

**Published:** 2025-05-10

**Authors:** Sai Balakavi, Vineet Vadrevu, Kristofer Lasko

**Affiliations:** 1 Universities Space Research Association (USRA) Science and Technology Institute, Huntsville, AL 35805, USA; 2 Earth System Science Center, University of Alabama at Huntsville, Huntsville, AL 35805, USA; 3 James Clemens High School, Madison, AL 35756, USA; vineetvadrevu@gmail.com; 4 Geospatial Research Laboratory, Engineer Research and Development Center, US Army Corp of Engineers, Alexandria, VA 22315, USA; kristofer.d.lasko@erdc.dren.mil

**Keywords:** satellite remote sensing, very high resolution (VHR), PlanetScope, deep learning, UNET, GRU, burnt areas

## Abstract

Remote sensing is essential for mapping and monitoring burnt areas. Integrating Very High-Resolution (VHR) data with medium-resolution datasets like Landsat and deep learning algorithms can enhance mapping accuracy. This study employs two deep learning algorithms, UNET and Gated Recurrent Unit (GRU), to classify burnt areas in the Bandipur Forest, Karnataka, India. We explore using VHR imagery with limited samples to train models on Landsat imagery for burnt area delineation. Four models were analyzed: (a) custom UNET with Landsat labels, (b) custom UNET with PlanetScope-labeled data on Landsat, (c) custom UNET-GRU with Landsat labels, and (d) custom UNET-GRU with PlanetScope-labeled data on Landsat. Custom UNET with Landsat labels achieved the best performance, excelling in precision (0.89), accuracy (0.98), and segmentation quality (Mean IOU: 0.65, Dice Coefficient: 0.78). Using PlanetScope labels resulted in slightly lower performance, but its high recall (0.87 for UNET-GRU) demonstrating its potential for identifying positive instances. In the study, we highlight the potential and limitations of integrating VHR with medium-resolution satellite data for burnt area delineation using deep learning.

## 1. Introduction

Vegetation fires are a recurring phenomenon, driven by both natural factors and human activities such as land clearing, agriculture, and slash-and-burn practices [1]. These fires are most prevalent in tropical regions, particularly in South and Southeast Asian (S/SEA) countries, including in the Mediterranean, parts of Africa, and the Americas, where the combination of dry conditions and dense biomass creates ideal conditions for widespread burning [2]. In addition, most of the fires in these regions are driven by human factors. The impacts of vegetation fires in Asia are far-reaching [3]. They contribute significantly to greenhouse gas emissions, including carbon dioxide and methane, exacerbating global climate change [4,5]. Locally, these fires result in severe air pollution, often creating dense haze that affects millions of people, leading to respiratory and cardiovascular health issues [6]. Additionally, fires cause substantial damage to ecosystems, leading to biodiversity loss, habitat destruction, and degradation of soil quality [7,8]. The socio-economic repercussions are equally profound, with disruptions to agriculture, tourism, and livelihoods in affected areas. Thus, studies on mapping and monitoring of fires and addressing the impacts, gain significance in different regions of the world, including in S/SEA [9,10].

Remote sensing is vital for mapping and monitoring burnt areas, providing timely and cost-effective data over large regions [11]. Multispectral sensors, such as those on Landsat and Sentinel-2 satellites, are highly useful to detect burn scars using indices like the Normalized Burn Ratio (NBR) [12]. Thermal sensors in satellites such as Moderate Resolution Imaging Spectroradiometer (MODIS) and Visible Infrared Imaging Radiometer Suite (VIIRS) effectively capture active fire hotspots and fire radiative power to estimate fire intensity [13]. Further, time series data from these data facilitate monitoring fire progression and post-fire recovery; examples include the global products like the MODIS Burned Area Product (MCD64A1) offering standardized burnt area maps [14,15].

Compared to medium-resolution data such as Landsat or Sentinels, Very High-Resolution (VHR) data offers significant advantages [16,17]. The fine spatial detail of VHR data allows for the detection of small-scale burn scars and heterogeneous fire patterns, including patchy or low-intensity burns that are often missed by coarser datasets. The enhanced spatial accuracy is crucial for delineating fire perimeters with high precision, especially in fragmented landscapes or urban–wildland interfaces [18]. By minimizing mixed-pixel effects where a single coarse-resolution pixel may represent a blend of burned and unburned land cover types, the VHR imagery significantly improves classification precision, leading to more reliable and detailed maps of burn extent, which are critical for accurate reporting, resource allocation, and response planning. Further, beyond initial burn detection, VHR data can also support high-resolution post-fire assessments. Their granularity can enable analysts to identify specific fire impacts on individual agricultural plots [16], buildings, road networks, and small vegetation patches [19]. Such insights are particularly valuable in areas where wildfires directly affect human settlements, infrastructure, or livelihoods. Moreover, the detailed temporal and spatial information captured by VHR imagery can enhance the assessment of fire severity and facilitate monitoring of post-fire vegetation recovery at a micro-scale, offering key inputs for ecological studies and long-term land management.

However, despite the advantages of VHR, the use of these data is not without challenges. VHR datasets are often expensive and cover smaller geographic areas, making them less suitable for large-scale monitoring compared to medium or coarse-resolution data. Integrating multi-satellite data for environmental monitoring in general, and burnt areas in particular, is a promising approach [20] for accurate and scalable burnt area mapping, especially when leveraging machine learning and deep learning techniques. In particular, deep learning algorithms, those utilizing transfer learning and multi-scale feature extraction, have immense potential for integrating VHR and medium-resolution data [21]. By using pre-trained models on large-scale VHR data and fine-tuning them on medium-resolution imagery such as Landsat or Sentinels, the rich feature representations learned from high-detail data is effectively transferred to medium-resolution or coarser datasets. Similarly, architectures like U-shaped Convolutional Neural Network (UNET) or its variations can be adapted to exploit the hierarchical feature relationships between the two resolutions, ensuring the models capture both localized and broader spatial patterns of burnt areas [22,23].

In this study, we employ two advanced deep learning algorithms—UNET [24] and Gated Recurrent Unit (GRU) [25]—to classify burnt areas in the Bandipur Forest, Karnataka, India. These models were selected for their ability to learn complex spatial and temporal patterns, critical for accurate burnt area detection. Initially, we evaluated the performance of each model using Landsat imagery, with training and testing data derived solely from the same medium-resolution satellite. This helped establish a baseline for comparison by assessing how each model performs when inputs and labels originate from the same sensor. Subsequently, we introduced a novel cross-sensor classification framework. In this approach, high-quality burnt area labels were derived from VHR PlanetScope imagery (3 m) and then used to train the models on the medium-resolution Landsat imagery (30 m) for classification. This setup allowed us to evaluate the ability of the models to generalize across spatial resolutions. VHR data enabled the generation of detailed, accurate labels with fewer samples, reducing the need for extensive field data collection. By leveraging these rich annotations, our method addresses a critical challenge in remote sensing, which often relates to the scarcity of reliable ground-truth data for model training and validation using mid-resolution remote sensing data.

Overall, our study introduces a novel cross-resolution classification framework that leverages high-resolution PlanetScope imagery to generate accurate burnt area labels, which are then used to train deep learning models for classifying medium-resolution Landsat data. We tested the potential contributions of both UNET and GRU architectures in this cross-resolution setting for achieving high classification accuracy. Our cross-scale resolution mapping strategy offers a valuable alternative to conventional methods, particularly in data-scarce or resource-constrained environments. By utilizing high-resolution PlanetScope imagery to generate accurate burnt area labels and applying these labels to train models on medium-resolution Landsat data, our approach effectively bridges the resolution gap. The implications of this study extend beyond wildfire monitoring, providing a framework for applying high-resolution annotations to classify medium-resolution datasets in various environmental monitoring applications.

## 2. Study Area

**Figure 1:**
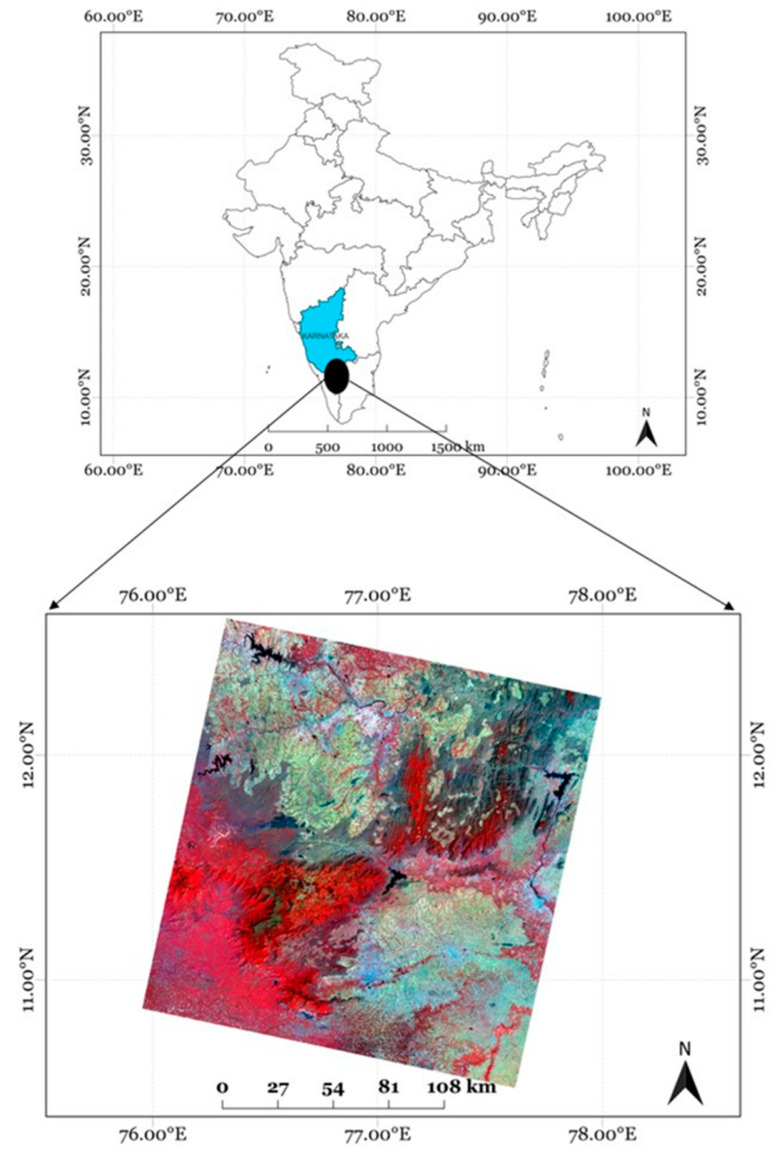
Location map of the Bandipur Forest area, Karnataka, India. The raw Landsat image, corresponding to 29 February 2019, is also shown.

Bandipur Forest, located in the southern Indian state of Karnataka, stands as one of the most prominent national parks and tiger reserves in the region Figure 1. It forms an integral part of the Nilgiri Biosphere Reserve, which is globally recognized for its exceptional biodiversity hotspot [26]. The national park is home to a wide range of flora and fauna. Its diverse ecosystems are vital for maintaining ecological balance in the region, serving as a critical wildlife corridor that supports the movement and survival of many species. Bandipur faces increasing threats from forest fires due to human activities, especially during the dry season, posing a serious challenge to the park’s delicate ecosystems [26]. Addressing fires in the national park requires advanced monitoring and management strategies to mitigate the negative impact of fires on the forest’s biodiversity. High-resolution satellite data combined with the latest and robust algorithms can provide valuable information for understanding the extent and impact of forest fires in the park, contributing to more effective conservation efforts.

## 3. Methods

In this study, we tested two different deep learning models, a custom UNET and custom UNET integrated with Gated Recurrent Unit (GRU) for mapping the burnt areas. Both models were tailored for semantic segmentation tasks and trained on 256 × 256-pixel image tiles with three bands: Near-Infrared (NIR), Red, and Green.

### 3.1. Data Sources

We used surface reflectance data from the Landsat 8 Operational Land Imager (OLI) dataset with a spatial resolution of 30 m, acquired on 25 February 2019, for the Bandipur study area, as shown in Figure 1. This dataset served as one of the primary sources for training and evaluation in this study. Landsat imagery is widely used in remote sensing due to its broad temporal availability and multispectral richness. This makes it suitable for applications such as land cover classification, environmental monitoring, and urban expansion studies. However, due to its coarser 30 m spatial resolution, it can present limitations when analyzing small or fragmented burnt patches that require finer spatial detail. This limitation motivated the cross-scale learning approach adopted in this work, where high-resolution data are used to augment and inform classification on medium-resolution data.

**Figure 2:**
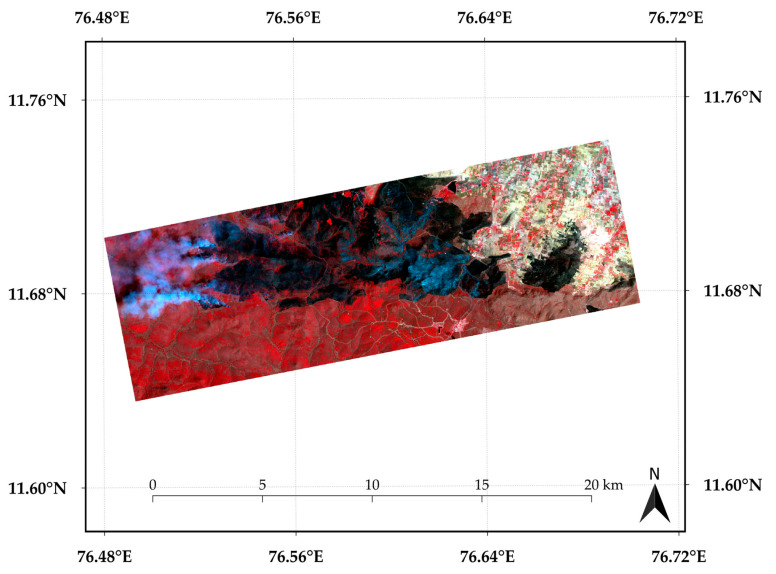
Location map of the Bandipur Forest area, Karnataka, India. The raw PlanetScope image, corresponding to 29 February 2019, is also shown. The black areas depict burnt areas.

For VHR reference, we used PlanetScope (3 m) data from the same date and study area as shown in Figure 2. Operated by Planet Labs, PlanetScope provides daily global imagery with a spatial resolution ranging from 3 to 5 m, enabled by a constellation of agile CubeSats. Its combination of high spatial and temporal resolution makes it highly effective for tasks like burnt area mapping, land use classification, agricultural monitoring, and disaster assessment.

In this study, we manually generated burnt area labels using both PlanetScope and Landsat imagery. These two sets of labels were then applied to the Landsat imagery for training and evaluating the deep learning models. This dual labeling approach allowed us to compare how high-resolution and medium-resolution labels affect model performance when applied to medium-resolution input data.

By evaluating models trained with PlanetScope-derived labels against those trained with Landsat-derived labels, both applied on Landsat imagery, we assessed the effectiveness of very high-resolution labeling for improving burnt area classification on coarser-resolution satellite data.

### 3.2. Label Generation

Our study focused on binary classification of burnt versus unburnt areas using deep learning algorithms. Due to the use of VHR PlanetScope imagery (3 m), burnt regions were visually distinguishable as dark patches, which helped reduce the ambiguity commonly associated with mixed pixels in the medium-resolution imagery. To ensure accurate labeling and focus exclusively on burnt areas over forests, we first derived a forest mask from a 10 m resolution Sentinel-2 land cover product. All the non-forest classes, including water bodies, roads, urban areas, and barren land, were excluded to derive the forest mask. This forest mask was then used to streamline the accuracy of the burnt area label generation process and reduce potential misclassification with other non-forest classes. With the forest mask applied, we manually digitized burnt areas in QGIS using visual interpretation of the PlanetScope imagery. A shapefile containing burnt areas was created and used to extract representative samples from the masked image. A careful selection process ensured high accuracy in the chosen samples, targeting clear and homogenous regions for delineating the burnt and unburnt areas. Further, the shapefile was processed using QGIS’s Sample Raster Values tool, allowing us to extract spectral values across all four PlanetScope bands (Red, Green, Blue, and NIR) for the sampled burnt areas. Minimum and maximum values for each band were calculated using a multi-band thresholding approach using the raster calculator in QGIS to generate a high-resolution (3 m) binary burnt area mask useful for model training and evaluation. In addition to the PlanetScope-derived labels, we also generated a second set of labels using Landsat imagery (30 m resolution). The same forest mask was applied to the Landsat data to ensure consistency. To assist in identifying burnt areas on Landsat, the manually digitized PlanetScope burnt areas were overlaid and used for cross verification. Then, PlanetScope- and Landsat derived-labels were used independently to train models and evaluate classification performance on the input Landsat (30 m) imagery. This dual-labeling strategy enabled a comparative analysis of how label resolution (high-resolution vs. medium-resolution) affects the segmentation performance in a cross-resolution learning setup.

### 3.3. Custom UNET Architecture

**Figure 3:**
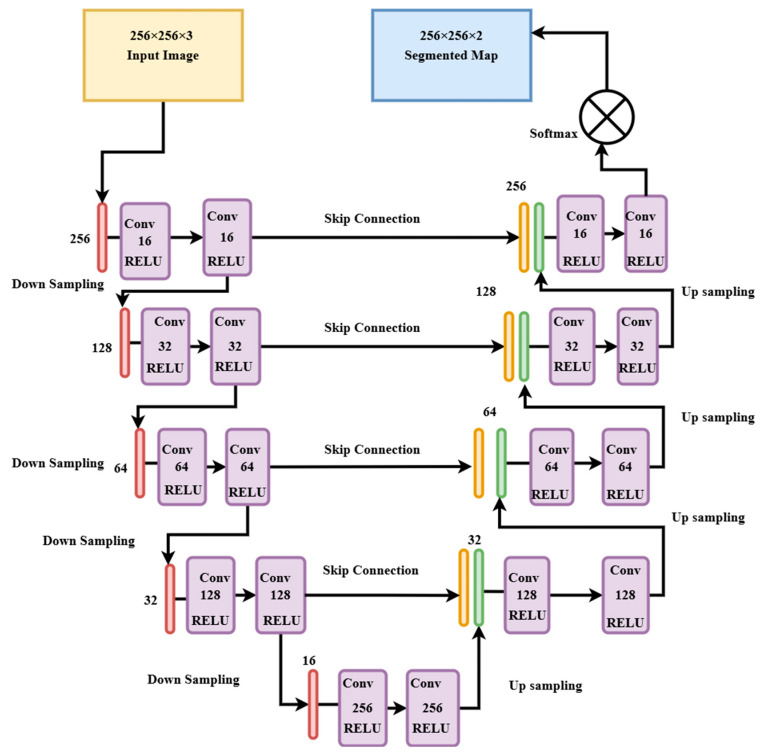
Custom UNET architecture used in the training process depicting the encoder, decoder, filters, and activation functions used.

The architecture implemented in this study is inspired by the base UNET model, widely used for semantic segmentation tasks in biomedical and remote sensing areas [27,28]. It consists of a symmetric encoder–decoder structure with skip connections. The encoder includes four convolutional blocks, each with two 3 × 3 convolutions followed by Rectified Linear Unit (ReLU) activation [29], 2 × 2 max-pooling, and a dropout layer with 0.2 (20%) to reduce overfitting. Filters double at each block, starting from 16 to 256 at the bottleneck, to capture all the complex features, as shown in Figure 3.

The decoder mirrors the encoder, using transposed convolutions for the upsampling path. The skip connections help to recover spatial detail. The final segmentation map is generated using a 1 × 1 convolution followed by SoftMax activation. The model was trained using the Adam optimizer with a learning rate of 1 × 10^−4^ and categorical cross-entropy loss. Model Checkpoint and ReduceLROnPlateau callbacks were used to retain the best-performing model and fine-tune the learning rate during training.

### 3.4. Custom UNET-GRU

**Figure 4:**
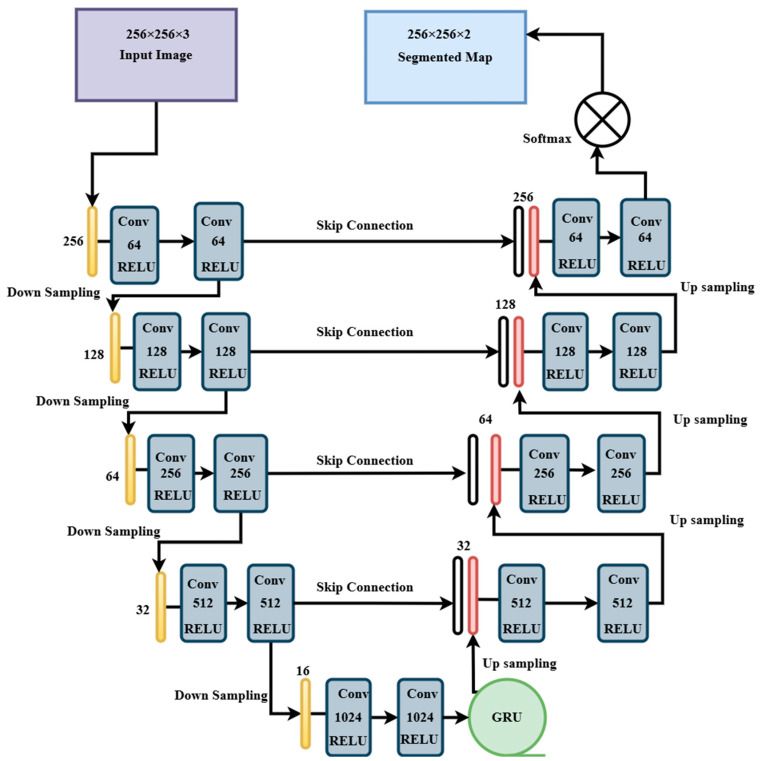
Custom UNET-GRU architecture used in the training process with encoder, decoder, filters, and activation functions used.

UNET-GRU, which integrates a GRU layer [30,31] at the bottleneck, was proposed to improve spatial context modeling. The encoder is similar to the UNET but uses deeper convolutional filters, starting from 64 and reaching 1024 at the bottleneck, as shown in Figure 4. After reshaping the encoded features, a GRU layer with 512 units models spatial dependencies across the 2-dimensional structure before reshaping the features back to spatial dimensions for decoding.

The decoder is similar to UNET, with transposed convolutions and skip connections, reducing filters from 512 to 64 across four upsampling blocks. A 1 × 1 convolution and SoftMax activation yield the final segmentation map. Training procedures, loss function, and callbacks are similar to those used in the UNET model.

### 3.5. Summary of Architectures

Even when both the models follow a fully convolutional encoder–decoder framework, the key architectural differences influence their performance and generalizability. The custom UNET model uses a standard convolutional structure with symmetric encoder and decoder paths, while the UNET-GRU model introduces a GRU layer at the bottleneck to enhance the retention of spatial dependencies. The UNET-GRU model also uses deeper convolutional blocks to capture more abstract features. Table 1 summarizes the core distinctions between the two models, including input configurations, filter sizes, GRU integration, loss functions, and training strategies.

### 3.6. Testing and Validation

The performance of the proposed models, the custom UNET and UNET-GRU, was evaluated through comprehensive testing and validation procedures. Both of these models were evaluated on metrics like Accuracy, Mean Intersection over Union (IoU), F1-Score, Precision, Recall, and a Confusion Matrix. These metrics provided a detailed understanding of how well each model performed on unseen data, while the validation step further reinforced their generalization capabilities across diverse image features. Also, these metrics provided a multidimensional assessment of the models’ effectiveness in leveraging high-resolution PlanetScope-derived labels on lower-resolution Landsat imagery, compared to the original Landsat labels.

### 3.7. Data Augmentation and Evaluation

A data augmentation technique was used to enhance the robustness of the training process. Image generator was used to create batches of augmented validation images along with their corresponding burnt area masks. This technique helped the models to generalize better by introducing randomness in the validation data. Predicted masks and the images were processed to extract the respective class labels, which were then used to calculate the metrics.

Our current study focused on binary burnt vs. unburnt classification using various deep learning methods. Thus, it was easy for us to avoid mixed pixel cases with all land cover types. The labeled data on burnt and un-burnt areas patches came from the PlanetScope data, which are of 3 m resolution. With this high resolution, the burnt areas were clearly visible as black patches on the PlanetScope image.

To focus the analysis only on forested regions and eliminate ambiguity from other land cover types such as water bodies, roads, urban areas, and barren land, we derived a forest mask from a 10 m resolution Sentinel-2 land cover classification product. All non-forest classes were excluded. This helped to focus on the forested areas only. This forest mask was used to spatially constrain the label generation process and minimize misclassification due to spectral overlap with non-target land cover types.

A new shapefile with burnt area polygons was created in the QGIS for burnt and un-burnt areas from the PlanetScope image with a forest mask on it. This shapefile helped to collect numerous precise samples across the masked PlanetScope image. A meticulous selection process was employed to make sure there was high accuracy in the picked PlanetScope samples. After collecting the samples, the shapefile was processed using the Sample Raster Values feature of the QGIS. This feature allowed us to analyze the raster values at the selected points and played a crucial role in determining the characteristics of the PlanetScope-derived burnt area patches. Following this step, the minimum and maximum from each band were noted. Then, a thresholding technique was applied with these values in the Raster Calculator of the QGIS, which generated the burnt areas mask from PlanetScope data, which served as a reliable VHR-derived burnt area label. This mask was further used for training the model using both the PlanetScope as well as Landsat data to generate accurate segmented results for the burnt areas.

Independent of this exercise, we also created labeled data using the Landsat imagery with the forest mask applied and used them for classification with the same algorithms, i.e., the custom UNET and UNET-GRU. When creating labels using the Landsat imagery, the original PlanetScope labels were overlaid on the Landsat imagery for cross-checking to identify the burnt area patches on the Landsat imagery. We performed a comparative model performance exercise separately, with models trained on PlanetScope-derived labels versus Landsat-derived labels when evaluated on the Landsat imagery.

We report the classification results for the Landsat-derived burnt areas for the four different cases, as follows: (a) custom UNET classification with Landsat labels and the resulting classification; (b) custom UNET classification with PlanetScope labeled data applied on Landsat; (c) custom UNET-GRU classification with Landsat labels and the resulting classification; (d) custom UNET-GRU classification with PlanetScope labeled data applied on Landsat.

This comparative setup enabled us to assess the impact of label resolution on model performance, assess the architectural advantages of UNET-GRU over the baseline UNET. Evaluation metrics included Accuracy, Precision, Recall, F1-Score, Intersection over Union (IoU), and confusion matrix analysis. The results identified the best classification algorithm, as well as the use of Landsat- and PlanetScope-labeled data on Landsat-derived burnt areas. Table 2 provides the distribution of tiles used in each split across PlanetScope and Landsat imagery.

## 4. Results

To evaluate the model performance, we used standard classification and segmentation metrics like Precision, Recall, F1-Score, Accuracy, Mean Intersection over Union (IoU), and Dice Coefficient. The definitions for these metrics are as follows: Precision measures the percentage of correctly predicted burnt pixels among all the predicted burnt pixels, which indicates how well the model avoids false positives. Recall captures the proportion of actual burnt pixels that were correctly identified, which indicates the model’s sensitivity toward the target class. F1-Score is the harmonic mean of precision and recall, balancing both accuracy and coverage. Accuracy is the overall percentage of correctly classified pixels. IoU evaluates the overlap between predicted and actual burnt regions in pixel-wise segmentation. The Dice Coefficient, similar to the F1-Score, quantifies the segmentation quality, where higher values indicate a better spatial match.

**Figure 5:**
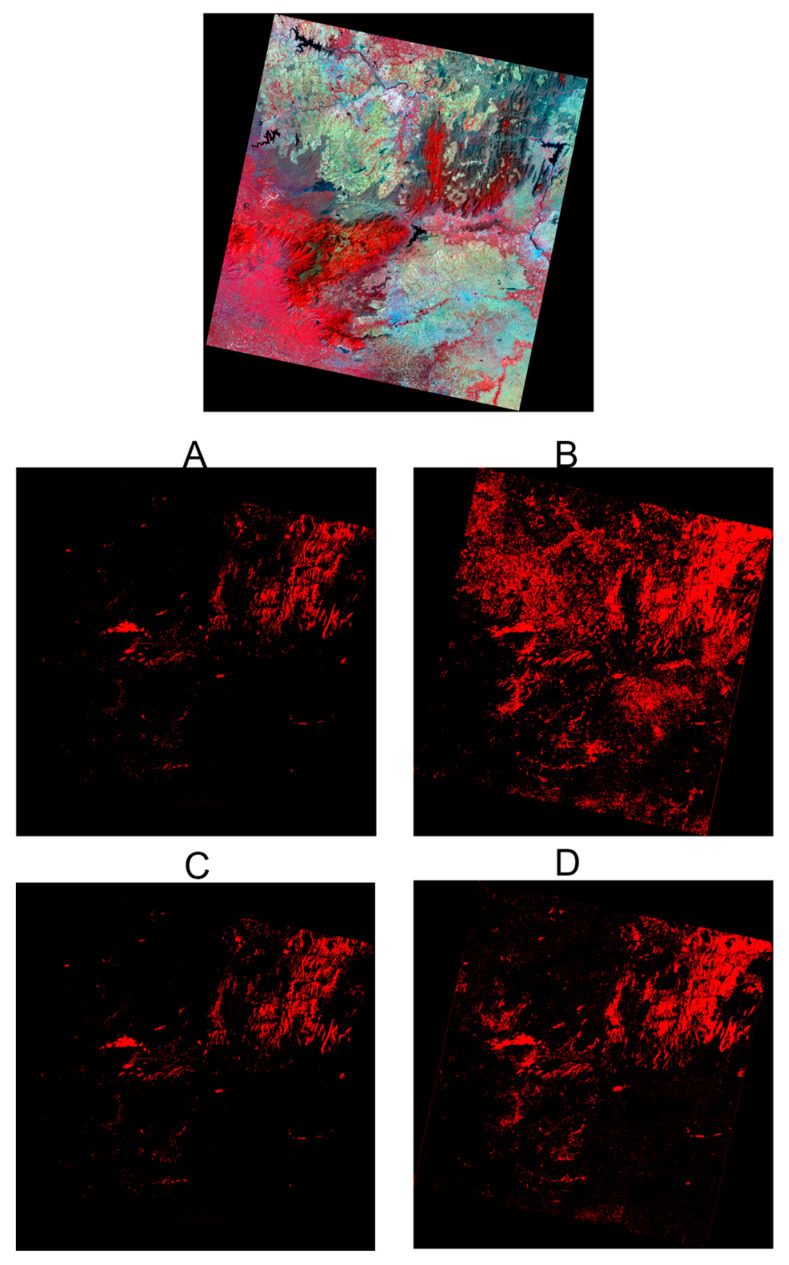
UNET and GRU models were used to map burnt areas in Landsat data. The top image shows raw data over Bandipur National Park, India, from 25 February 2019. Burnt area mapping results from four cases are depicted, with burnt areas in red and unburnt areas in black: (**A**) UNET model with Landsat-derived labels, (**B**) custom UNET model with PlanetScope-labeled data, (**C**) custom UNET-GRU model with Landsat labels, and (**D**) custom UNET-GRU model with PlanetScope-labeled data. Among these, the custom UNET model with Landsat labels performed best, achieving a precision of 0.89, accuracy of 0.98, and segmentation quality scores of Mean IoU: 0.65 and Dice Coefficient: 0.78.

The performance of the custom UNET and UNET-GRU models was assessed on Landsat imagery using two labeling strategies, one derived from Landsat itself and the other from PlanetScope. Figure 5 presents the classified outputs, and the metrics are summarized in Table 3. When trained with Landsat-derived labels, the custom UNET model achieved the highest precision and accuracy, reflecting strong segmentation quality with minimal false positives. In contrast, the custom UNET-GRU model trained with PlanetScope-derived labels demonstrated the highest recall value of 0.87, indicating that the model captured more of the burnt area but at the cost of lower precision This trade-off arises from the finer spatial resolution of PlanetScope (3 m) labels, which helps with the detection of smaller and fragmented burnt areas but also increases the chances of false positives. Overall, the models using Landsat-derived labels performed better in terms of F1-Score and IoU, indicating a stronger balance between accuracy and pixel-wise segmentation.

### 4.1. Custom UNET Trained on Landsat Labels Applied on Landsat

This approach performed well across most metrics, making it one of the top models in the table. It has a high Precision of 0.89, indicating that when it predicts a positive instance, it is highly likely to be correct, with fewer false positives. Its Recall is 0.57, meaning that it identifies a decent portion of the positive instances, though it misses some positives, resulting in a moderate rate of false negatives. The F1-Score of 0.78 reflects a solid balance between precision and recall, showing that the model strikes a good trade-off between minimizing both false positives and false negatives. In terms of Accuracy, the custom UNET model trained on Landsat labels scored an impressive 0.98, meaning that it correctly classifies a high proportion of both positive and negative instances. This indicates that the model performs exceptionally well overall, even though its recall could be improved. The Mean IoU of 0.65 indicates that the model has a strong overlap between its predicted segmentation and the burnt area mask, performing well in accurately identifying the regions of interest. Similarly, the Dice Coefficient of 0.78 shows that the model’s segmentation quality is good, with a strong similarity between the predicted and actual segmented areas. Overall, this approach stands out for its solid performance in precision, accuracy, and segmentation, making it one of the best models in this comparison.

### 4.2. Custom UNET Trained on PlanetScope Labels Applied on Landsat

This approach performed weakly across most of the evaluation metrics. Its Precision is very low at 0.18, indicating that when it predicts a positive instance, there is a high likelihood that it is incorrect, leading to many false positives. Despite this, the model has a relatively high Recall of 0.81, meaning it successfully identifies most of the positive instances, though it still misclassifies many negatives as positives. The F1-Score of 0.29 reflects a poor balance between precision and recall, showing that while recall is high, the low precision significantly impacts the overall performance. In terms of Accuracy, this approach has a relatively low score of 0.63, indicating that it struggles with correct predictions overall. The Mean IoU is only 0.17, suggesting that the model has very poor segmentation overlap with the burnt area mask, failing to effectively capture the regions of interest. Similarly, the Dice Coefficient of 0.29 indicates that the model performs poorly in terms of segmentation quality, with a weak overlap between the predicted and actual segmented areas. Overall, custom UNET classification with PlanetScope labeled data applied on Landsat demonstrated weak performance across all metrics, particularly in terms of precision and segmentation quality, making it the least effective model in this comparison.

### 4.3. UNET-GRU Trained on Landsat Labels Applied on Landsat

This approach demonstrates strong performance in several key metrics, making it one of the top models. Its Precision is very high at 0.97, meaning that when the model predicts a positive instance, it is almost always correct, with very few false positives. However, its Recall is moderate at 0.62, indicating that while the model identifies a good portion of the positive instances, it still misses some, leading to a higher number of false negatives compared to its precision. The F1-Score of 0.75 reflects a decent balance between precision and recall, showing that the model effectively balances the need to minimize both false positives and false negatives. In terms of Accuracy, the UNET-GRU model excelled with a score of 0.98, suggesting that it correctly classifies most positive and negative samples, making it a reliable model overall. The Mean IoU of 0.6 indicates that the model has a good overlap with the burnt area mask in terms of segmentation, though there is room for improvement. The Dice Coefficient of 0.75 further confirms the model’s strong segmentation performance, showing that it produces relatively accurate and similar segmentation results compared to the true values. Overall, this approach performs very well, especially in terms of precision, accuracy, and segmentation quality, with a good balance between identifying positive instances and maintaining low false positives.

### 4.4. UNET-GRU Trained on PlanetScope Labels Applied on Landsat

This approach exhibited mixed performance across the evaluation metrics, showing both strengths and weaknesses. Its Precision is relatively low at 0.49, meaning that while it identifies some positives correctly, it also produces a significant number of false positives. However, its Recall is quite high at 0.87, indicating that the model successfully captures most of the positive instances, even though this comes at the cost of more false positives. The F1-Score of 0.63 reflects this imbalance, indicating that while recall is strong, the low precision negatively impacts the model’s overall performance. Accuracy is solid at 0.95, suggesting that the model makes correct predictions most of the time. However, the high recall, coupled with low precision, leads to a less balanced prediction outcome. The Mean IoU of 0.46 suggests that the model’s segmentation performance is decent but not outstanding, with a moderate overlap between the predicted and true regions. Similarly, the Dice Coefficient of 0.63 indicates a reasonable level of segmentation accuracy, though it could still be improved. Overall, UNET-GRU classification with PlanetScope labeled data applied on Landsat performed well in terms of recall and accuracy but struggled with precision, leading to a lower F1-score and moderate segmentation performance.

Based on the above evaluation metrics, among the four models, we infer that the custom UNET with Landsat labels had the best model performance overall, excelling in precision (0.89), accuracy (0.98), and segmentation quality (Mean IoU: 0.65, Dice Coefficient: 0.78). Its solid balance between precision and recall (F1-Score: 0.78) makes it the top performer overall. The second-best model is UNET-GRU with Landsat labels, which also demonstrates strong performance with high precision (0.97) and accuracy (0.98), although its recall (0.62) is lower, resulting in a slightly lower F1-Score (0.75). The third best is UNET-GRU with the PlanetScope labels, which excels in recall (0.87), but its low precision (0.49) and moderate segmentation performance (Mean IoU: 0.46, Dice: 0.63) pull down its overall F1-Score (0.63). Finally, custom UNET with the PlanetScope labels ranks fourth, with very low precision (0.18) and a poor F1-Score (0.29), indicating it performs poorly in both classification and segmentation tasks.

The results in terms of image pixels and percentages comparing the performance of different deep learning models—Custom UNET and Custom UNET-GRU—trained on Landsat and PlanetScope labels are given in Table 4. The predictions vary significantly. The Custom UNET with PlanetScope labels overestimated the burnt area, predicting 22.19% compared to the actual 4.4%, leading to the highest misclassification rate of 17.45%. In contrast, the Custom UNET with Landsat labels and the Custom UNET-GRU with Landsat labels demonstrated the most accurate performance, with misclassification rates of 1.62% and 1.79%, respectively. The Custom UNET-GRU with PlanetScope labels had an intermediate misclassification rate of 4.59% but still overestimated the burnt area at 7.87%. Overall, the Custom UNET-GRU model trained with Landsat labels achieved the best performance, minimizing misclassifications while closely matching the burnt area mask.

To further interpret the performance differences across all the model-label combinations, we analyzed how model architectures and label resolutions influence the segmentation outcomes. The custom UNET model trained on the Landsat-derived labels delivered the strongest overall performance. This is likely due to the close spatial and spectral alignment between the input and training data, both originating from the same sensor Landsat 8 OLI. In contrast, the UNET-GRU model trained on PlanetScope labels achieved the highest Recall (0.87), indicating its ability to capture small and fragmented burnt areas more effectively. However, this improvement came at the cost of increased false positives, which shows the sensitivity–precision trade-off. This behavior reflects the model’s responsiveness to high-resolution supervision, even when applied to lower-resolution imagery. Conversely, the UNET model trained on PlanetScope labels showed the weakest performance. These results suggest that architectural capacity and resolution compatibility can play a key role in enabling generalization across sensor domains.

The above results also illustrate the practical and methodological challenges introduced by resolution mismatch between PlanetScope (3 m) training labels and Landsat (30 m) input imagery. While higher-resolution labels provide the critical spatial detail, they also risk introducing label noise when aggregated or projected onto coarser grids. However, our findings show that high-resolution PlanetScope masks can still provide valuable supervision for medium-resolution models when they are carefully sampled and spectrally constrained. The use of VHR data such as PlanetScope is especially effective when the samples are further refined using a forest mask to exclude non-target land cover types, as in our case. Integration of VHR data is a viable path for fire monitoring in areas where such data are sporadic, but medium-resolution datasets like Landsat (30 m) are abundant and temporally rich.

## 5. Discussion

Overall, the models demonstrated strong performance when evaluated with Landsat-derived labels, with custom UNET consistently outperforming custom UNET-GRU across most metrics. This superior performance highlights the inherent compatibility between Landsat labels and Landsat imagery, as both share the same spatial resolution. This alignment reduces discrepancies in spatial and spectral details, enabling the models to produce reliable results. The high accuracy of predictions confirms the effectiveness of using Landsat-derived labels for training models, making it a dependable choice for applications requiring consistent spatial resolution.

When labels from PlanetScope were used to classify the Landsat data, the models showed a drop in performance compared to those trained with Landsat labels. For instance, the custom UNET-GRU model achieved a Precision of 0.49 and an F1-Score of 0.63, while the custom UNET model had a Precision of 0.18 and an F1-Score of 0.29. These results illustrate the challenges of adapting high-resolution labels to medium-resolution imagery, where fine spatial detail may be lost. However, the consistently high Recall scores (0.87 for UNET-GRU and 0.81 for UNET) suggest that PlanetScope-derived labels enable models to detect a broader range of burnt features, even if precision and segmentation quality are lower.

Our PlanetScope derived labels were generated using a multi-band thresholding using nir, red, green, and blue bands with a method based on spectral values. The labels were carefully sampled over the burnt regions. Further, we used a Sentinel-2 data-derived forest mask to exclude other land cover classes, as our focus was on burnt area mapping. Although the forest mask helped reduce false positives from roads, water, and non-vegetated surfaces, future work could benefit from adaptive thresholding [32], object-based classification, or self-supervised labeling methods [33] to improve performance in spatially heterogeneous landscapes.

Several studies have demonstrated the value of multi-sensor integration and transferring of labels for improved fire classification. For instance, Ref. [34] used Sentinel-2 and MODIS fusion to improve burnt area detection, while [35] employed thresholding and manual validation techniques with Sentinel-2 to prepare labels for UNET-based classifiers. Ref. [36] highlighted the value of UNET segmentation using Landsat time series, achieving a Dice score of 0.93. Similarly, Ref. [37] applied spatial-temporal deep learning models on Landsat for global-scale burnt area mapping. Ref. [38] emphasized the importance of spatial alignment when combining MODIS and Landsat data for agricultural monitoring, highlighting the challenges and opportunities in cross-resolution analysis that parallel our approach. Ref. [39] introduced a semi-supervised framework leveraging PlanetScope and Sentinel-2 to address data scarcity in fire monitoring. The fusion of VHR and Sentinel-2 imagery has been shown to effectively capture vegetation disturbances, supporting our approach of incorporating fine spatial detail to enhance classification outcomes [40]. In line with this, a recent cross-resolution framework employing a patch-based supervision and contrastive learning approach further validates the feasibility of training medium-resolution models using sparse VHR labels [41].

In addition to these, transfer learning strategies have emerged as a promising solution for multi-sensor challenges. For example, Ref. [42] used transfer learning with multi-sensor fire smoke detection data to enhance generalization, and [43] demonstrated the effectiveness of cross-sensor feature representation in early fire detection scenarios. Meanwhile, Ref. [44] improved segmentation by combining Landsat with Visible Infrared Imaging Radiometer Suite (VIIRS) hotspot data, reinforcing the importance of integrating detection and validation sources. These studies show that cross-resolution learning strategies can be effective when carefully adapted to spatial, temporal, and spectral properties.

Other traditional classification methods remain effective as well. For example, Ref. [45] demonstrated strong performance using Random Forest classifiers in image-based fire detection, while [46] achieved high accuracy in fire type classification using high-resolution deep learning models. Additionally, Ref. [11] developed the MCD64A1 MODIS burned area product, a widely accepted benchmark in operational fire monitoring.

Our label generation strategy aligns with the above highlighted studies in its use of open-source GIS tools, spectral thresholds, and cross-resolution consistency. Our method retains unique pixels and manually verifies thresholds using both PlanetScope and Landsat imagery. This approach enhances adaptability, transparency, and interpretability, particularly in heterogeneous or data-scarce regions. Further, the high recall achieved using PlanetScope labels suggests that fine-grained features contribute to better detection, while Landsat-derived labels offer better segmentation boundaries due to resolution alignment. This complementarity mirrors findings in other multi-sensor research and supports a hybrid workflow. The combination of PlanetScope’s revisit frequency and spatial detail with Landsat’s stability and precision allows for accurate, timely burnt area classification.

Despite these strengths, our study is limited by its reliance on a single source of PlanetScope and Landsat imagery. Future research could incorporate multi-temporal data to map burnt area transitions over time. However, despite this limitation, our study proved valuable in identifying a novel deep learning-based algorithm and analyzing variations in derived burnt area labels for cross-sensor classification. In conclusion, our approach provided valuable insights into a range of deep learning algorithms and facilitated a cross-sensor comparative study on burnt area delineation. By integrating robust label generation, interpretable segmentation models, and complementary satellite data, our study established a scalable and transferable framework for burnt area mapping in the study region. This methodology not only aligns with current research trends but also offers practical tools for advancing multi-resolution remote sensing applications in other regions.

In summary, this study introduced a novel cross-resolution training framework by integrating very high-resolution PlanetScope (3 m) data with medium-resolution Landsat (30 m) imagery for burnt area classification. Resolution-bridging supervision is a novel approach that remains largely untapped in the fire mapping applications. The demonstrated ability to use sparse VHR samples to guide classification on widely available medium-resolution data offers a scalable and practical solution for fire monitoring in resource-constrained settings.

## 6. Conclusions

The comparative performance analysis of four models highlights distinct differences in the effectiveness of Landsat and PlanetScope data labels for burnt area delineation. The custom UNET model trained with Landsat labels emerged as the most effective, achieving the highest precision (0.91), accuracy (0.89), and segmentation quality (Dice Coefficient: 0.85). These results indicate its suitability for applications demanding high accuracy and reliable predictions. The UNET-GRU model also demonstrated strong performance, particularly in precision (0.87) and accuracy (0.86); however, its slightly lower recall (0.74) resulted in a reduced F1-score (0.80).

Conversely, the UNET-GRU model trained with PlanetScope labels exhibited strong recall (0.87) but lower precision (0.49), making it more appropriate for applications where maximizing the identification of positive instances is critical. In contrast, the custom UNET model with PlanetScope labels performed the worst across all metrics, particularly in precision (0.18) and segmentation quality (Dice Coefficient: 0.29).

We also infer the need to expanding our study and methods to larger areas and varied applications to fully understand the ability of various deep learning models, including integration of VHR and medium-resolution datasets for land use/cover classification in general and burnt areas in particular.

## Figures and Tables

**Table 1:** Summary of architectural differences between the custom UNET and custom UNET-GRU models used for burnt area classification.

Component	UNET	UNET-GRU
Input Size	256 × 256 × 3 (NIR, Red, Green)	256 × 256 × 3 (NIR, Red, Green)
Encoder	4 Conv blocks: ReLU + MaxPool	4 Conv blocks: ReLU + MaxPool
Filters (Encoder)	16 to 256	64 to 1024
Dropout	20% after each Conv Block	20% after each Conv Block
GRU Integration	Not applicable	GRU (512 units) after bottleneck
Decoder	4 upsampling blocks + skip connections	4 upsampling blocks + skip connections
Filters (Decoder)	256 → 16	512 → 64
Final Layer	1 × 1 Conv + SoftMax (2 classes)	1 × 1 Conv + SoftMax (2 classes)
Loss Function	Categorical Cross-Entropy	Categorical Cross-Entropy
Optimizer	Adam	Adam
Learning Rate	1 × 10^−4^	1 × 10^−4^
Evaluation Metrics	Accuracy, IoU, Precision, Recall, F1-score, ROC-AUC	Accuracy, IoU, Precision, Recall, F1-score, ROC-AUC
Weight Initialization	He Kaiming initialization	He Kaiming initialization

**Table 2:** Dataset composition and split allocation for PlanetScope and Landsat imagery used in model training and evaluation.

Category	PlanetScope	Landsat
Train	105	128
Test	17	21
Validation	18	22
Resolution	3 m	30 m

**Table 3:** Performance metrics for custom UNET and custom UNET-GRU deep learning models for burnt area delineation using Landsat data.

Model	Precision	Recall	F1-Score	Accuracy	Mean IoU
Custom UNET-GRU with Landsat labels	0.97	0.62	0.75	0.98	0.60
Custom UNET with PlanetScope labels	0.18	0.81	0.29	0.63	0.17
Custom UNET with Landsat labels	0.89	0.57	0.78	0.98	0.65
Custom UNET-GRU with PlanetScope labels	0.49	0.87	0.63	0.95	0.46

**Table 4:** Burnt area classification on Landsat data with different deep learning model combinations.

Metric	Custom UNET with Landsat Labels	Custom UNET with PlanetScope Labels	Custom UNET-GRU with Landsat Labels	Custom UNET-GRU with PlanetScope Labels
Burnt area pixels	2,585,869 (4.40%)	2,585,869 (4.4%)	2,585,869 (4.39%)	2,585,869 (4.39%)
Unburnt area pixels	56,222,696 (95.70%)	56,222,696 (95.61%)	56,222,696 (95.61%)	56,222,696 (95.61%)
Predicted burnt area pixels	1,820,952 (5.10%)	11,872,660 (22.19%)	1,621,529 (2.76%)	4,629,224 (7.87%)
Predicted unburnt area pixels	56,985,233 (96.90%)	46,933,505(79.82%)	57,184,636 (97.24%)	54,176,941 (92.13%)
Number of misclassified Pixels	952,072 (1.62%)	10,262,767 (17.45%)	1,051,218 (1.79%)	2,698,977 (4.59%)

## Data Availability

The original contributions presented in this study are included in the article. Further inquiries can be directed to the corresponding author.

## Abbreviations

The following abbreviations are used in this manuscript:

VHR	Very High Resolution
S/SEA	South and South East Asia
GRU	Gated Recurrent Unit
USRA	Universities Space Research Association
UAH	University of Alabama in Huntsville
